# Humanitarian forensic action in East Asia: where are we now? A concise review

**DOI:** 10.1093/fsr/owae014

**Published:** 2024-02-13

**Authors:** Zixuan Zeng, Yehui Lv

**Affiliations:** School of Basic Medical Sciences, Shanghai University of Medicine & Health Sciences, Shanghai, China; Institute of Wound Prevention and Treatment, Shanghai University of Medicine & Health Sciences, Shanghai, China; School of Basic Medical Sciences, Shanghai University of Medicine & Health Sciences, Shanghai, China; Institute of Wound Prevention and Treatment, Shanghai University of Medicine & Health Sciences, Shanghai, China

**Keywords:** forensic sciences, humanitarian forensic action, forensic humanitarianism, East Asia, review

## Abstract

Humanitarian forensic action (HFA) is practiced in many parts of the world. In recent years, with the development of forensic humanitarian methods and applications and the increasing inclusion or even prioritization of HFA by organizations and institutions, research in this field has evidenced greater depth and diversity, although perspectives from the humanities and some disciplines remain limited. In-depth inquiries into this topic and an analysis of regional humanitarian data reveal the existence of ideological and cultural foundations for HFA in East Asia. At the same time, given past occurrences of natural disasters and large-scale wars in this region, the need and motivation for advancing this field and developing HFA is considerable. Currently, because of a lack of practical experience of HFA in East Asia, research on this relevant topic in the region focuses on the development of humanitarian forensic applications. Consequently, studies reporting on social surveys, psychological care and other methods are limited. It is to be hoped that East Asian countries will improve their practical HFA applications, while simultaneously carrying out social surveys and social scientific research on all aspects of HFA.

**Key Points:**

- The current development of humanitarian forensic action is described in the field of different scientific disciplines and humanitarian applications in East Asia.
- The base of humanism of humanitarian forensic action in East Asia, and the necessity and feasibility of developing humanitarian forensic action are summarized.
- Lmitations of improving humanitarian forensic action in East Asia and ways to improve are presented.

## Humanitarian forensic action

Humanitarian forensic action (HFA) was first officially described by the International Committee of the Red Cross (ICRC) [1]. It is defined as the application of forensics to humanitarianism. From a historical perspective, it is evident that humanitarian action, such as providing medical care, food, and psychological care to the victims of natural and man-made disasters has always occurred. However, HFA, as a specific domain, only emerged during the previous century [2], marking a milestone in humanitarian action within contemporary society. HFA entails humane use of science to attend to humanitarian crises. The potential contribution of HFA is evident relative to the 2023 Turkiye–Syria earthquake, following the disappearance of Malaysia Airlines flight 370, and other events. Developments in multiple forensic disciplines, including anthropology, odontology, and especially genetics have increased our capacity to positively identify the dead. Our bibliometric study [3] of forensic humanitarianism revealed that social scientific studies are sparse. However, new perspectives are emerging for promoting the development of HFA within different scientific disciplines, such as the proposal to consider a community-grounded approach of forensic anthropology [4] and improving witness interview techniques in investigations of persons who have disappeared during armed conflicts [5]. This review offers insight on HFA from another perspective, enabling an expansion of the scope of discussion on HFA and bringing ethics, and psychological issues relating to HFA into focus.

## Different scientific discipline’s contribution to humanitarian forensic action

HFA is based on International Humanitarian Law (IHL) and is implemented within a framework of humanitarian care and legal support [6]. IHL mandates that both parties involved in a conflict handle the remains of the deceased in a dignified manner. It is based on these requirements that the search and identification of human remains through HFA become possible during wartime, enabling for more effective work in this regard. Furthermore, IHL provides humanitarian organizations in conducting HFA during times of war [7, 8]. It aims, for humanitarian reasons, to protect individuals who are not directly participating in hostilities, those who are no longer involved, the deceased, and their families. It also seeks to limit the means and methods of warfare. Compliance with IHL has facilitated the full utilization of various technical resources to support humanitarian efforts in times of war. Apart from legal considerations, there are also ethical factors involved in the development of HFA [9]. Informing the relatives of the deceased about the identity of their loved ones can be a complex and emotionally challenging task, particularly when injuries are involved, which is often the case [10]. One research direction in this field focuses on how to carry out this task without causing further harm. Those responsible for providing such information must be adequately prepared and sensitive to the circumstances, considering the potential pain it may inflict on the relatives of the deceased [11].

Mental health is another focus area in HFA research. With the progress of forensic humanitarianism, there is greater awareness for the need of research related to the complement-arity of psychology studies and HFA. For example, studies have been conducted on psychological evaluations of Guantanamo detainees [12] and the mental health and addictions of young people during the war in Ukraine [13, 14] show that the mental health needs of the population and the protection of the mental health of victims are also one of the focus areas in HFA. This is also in line with some of the current research in clinical forensics and clinical humanitarian forensics that can be combined to recognize the broader applicability of HFA [15].

**Figure 1:**
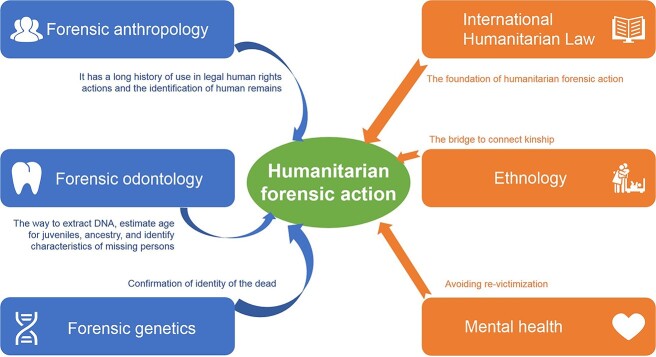
The primary roles of humanitarian applications and various scientific disciplines in humanitarian forensic action.

With the development of innovations in science and technology, applications for forensic humanitarianism are developing rapidly. Disciplines like forensic anthropology, forensic odontology, and forensic genetics have entered the mainstream worldwide. While their emphases differ, their associated methods can be deployed within HFA to analyse human remains, which is currently a focus area in forensic humanitarian work internationally (Figure 1).

Forensic anthropology has a longstanding history of being utilized in human rights investigations and for the identification of human remains. It draws upon the knowledge and methods of anthropology, specifically biological anthropology and archaeology, to address medico-legal issues. As a result, forensic anthropology is adept at approaching HFA from multiple angles, employing a range of techniques and methodologies to ensure scientifically sound outcomes [16]. These techniques and methods utilize technologies such as computed tomography [17], DNA recognition technology [18], imaging technology [19], artificial intelligence (AI) [20], and three-dimensional photogrammetry [21]. Through the use of these technologies and methods, human remains can be identified by characteristics such as ancestry, sex, time of death, and age [16]. With the ongoing development of new technologies, additional tools and approaches have been incorporated into the practice of forensic anthropology. As technological advancements continue and the field remains actively engaged, the application of forensic anthropology is bound to garner even more attention across various disciplines [22–25]. While the advancement of forensic anthropology has historically been concentrated in Europe and North America, research efforts in other regions are steadily increasing [16]. These studies aim to address gaps or limitations in ethnic, regional, and methodological investigations pertaining to forensic anthropology.

Forensic odontology is an effective discipline for identifying human remains. Given special dental conditions, such as the number and composition of teeth and the particularity of stomatology, forensic odontology has unique advantages for extracting DNA, age for estimating the age of juveniles, ancestry, and other identifying characteristics of missing persons [26]. Forensic odontology also has some unique advantages when applied in areas such as border control, human trafficking, or the identification of nameless cadavers [27]. At the same time, there is a certain subjectivity in the application of forensic odontology, which also enables the use of AI technology within forensic odontology [28]. Objective identification methods simplify the identification process to a certain extent while improving the credibility of identification by continuous training and peer review, which may also be a focus area within forensic odontology during the next stage of its development.

Forensic genetics provide relevant forensic evidence through genetic techniques. The sequencing of human genes within forensic genetics has been explored in a variety of ways, which include but are not limited to massively parallel sequencing (MPS), and representing the ability of sequencing technology to target a large number of different types of markers in the gene sequencing of human remains [29, 30]. Specific body fluids can be used for gene identification [31], while genetic information can be identified more accurately through the application of microhaplotypes [32]. These techniques indicate that human gene identification technology is advancing in the direction of high precision, multi-dimensional, and multi-scenic practices within forensic humanitarianism. Moreover, forensic humanitarian identification methods and tools can evidently be applied within nonhuman forensic genetics research [33]. For example, within zoology, the identification of the genetic characteristics, distribution, and diseases (e.g., Mad Cow Disease) of different animal species can improve the logical chain of forensic humanitarianism and provide reasonable evidence on-site for the investigation of a corpse present at a crime scene [34]. Within microbiology, evidence is provided by various microorganisms, such as *Bacillus anthracis*, HIV, SARS-CoV-2, and other specific microorganisms [35–37]. However, existing techniques in forensic genetics that are applicable to both humans and nonhumans have limitations. For example, it is difficult to preserve genetic information using techniques applied in human forensic genetics. There are still relatively few identifiable biogenesis techniques used in nonhuman forensic genetics [33]. However, there is no doubt that with the improvement of corresponding technical capabilities and the broadening of technical means, the application of forensic genetics in HFA will become increasingly advanced and comprehensive.

## The East Asian context

The inception of HFA is closely associated with external factors, such as the work of the Argentine Forensic Anthropology Team [2] and the International Commission on Missing Persons in former Yugoslavia [38]. East Asian countries (China, Japan, Republic of Korea (South Korea), Democratic People’s Republic of Korea (North Korea), and Mongolia) did not experience any large-scale wars and outflows of refugees and migrants during the period of HFA development; as such, relevant social organizations in East Asia were less involved in its development. Consequently, the development of HFA in East Asia is less advanced than in some other regions.

There are, however, some studies on humanitarianism in East Asia that have been conducted from the perspective of countries in this region. For example, studies were respectively conducted on the humanitarianism of medical staff in Japan [39] and nursing staff in China [40], and a third study focused on North Korean refugees in South Korea [41]. These studies are representative of humanitarian research conducted within East Asia. In addition, some humanitarian studies on East Asian countries have been conducted by researchers who are not from this region [42, 43]. These studies have objectively evaluated the humanitarian environment in East Asia from a third-party perspective. A review of studies conducted from both these perspectives shows that the humanitarian situation in East Asia differs from that in other regions. Humanitarianism in East Asia is more introspective and may not entail sufficient explicit actions, which may be the reason for limited HFA research conducted in this region.

Nevertheless, humanitarian action has been in evidence in the East Asian region over the past 2 500 years. Sun Wu’s *Sun Zi Bing Fa* (*The Ave of War*) noted the need to comfort wounded soldiers in China [44], while East Asian thought, represented by Confucianism, contains relevant content on “benevolence and righteousness”. Specifically, people are exhorted to sympathize with the weak and provide appropriate help [45], indicating that humanitarian action has some foundations in East Asia. In the present time, forensic humanitarian development in East Asia is progressing steadily through multidisciplinary efforts, technological improvements, and the establishment of various humanitarian organizations in East Asia. This progress is reflected in the fact that East Asian countries are beginning to pay attention to the application of forensic humanitarianism in rescue work associated with various disasters, which may be because HFA has a certain ideological basis in East Asia.

East Asian countries are prone to various natural disasters, such as earthquakes, tsunamis, and typhoons because of their unique geographical environments. These natural disasters result in large numbers of missing people [46], leading to a higher demand for HFA within East Asia. In addition, East Asia was one of the main war zones during the Second World War (WWII). In the 1950s, there were also major wars such as the Korean War. Millions of people died in the Asia-Pacific region due to WWII and the Korean War [47–50]. Studies have shown that in addition to the large-scale loss of life, these wars cause long-lasting trauma to survivors [51]. Hundreds of thousands of people went missing during these wars, highlighting the need for forensic humanitarianism to identify and deal with the problems created by these historical wars in East Asia from a humanitarian perspective. Ongoing efforts to search for soldiers involved in these conflicts are currently being conducted in China, South Korea, and other countries, reflecting the community’s commitment to HFA [52].

Furthermore, East Asian countries have diverse ethnic compositions, with China alone having 56 ethnic groups. The complex ethnic composition requires the development of forensic humanitarianism in East Asia and the implementation of HFA that are specific to this region. Several humanitarian institutions operate in East Asia. The ICRC Regional Delegation for East Asia, headquartered in Beijing, is responsible for ICRC’s humanitarian activities in the region. East Asian countries also have their own humanitarian organizations, such as the Red Cross Society of China, which has been in existence for nearly 120 years [53]. The further development of HFA in East Asia can be facilitated through collaboration between international and East Asian organizations.

## Humanitarian forensic action in East Asia

With the rising political status of East Asian countries on the world stage and the rapid advancement of science and technology in East Asia, some studies have been conducted on forensic humanitarianism in East Asia in recent years. These studies have focused on the development of humanitarian applications.

Ethnographic data in the field of forensic anthropology have expanded within East Asian countries, which are ethnically diverse. Several forensic anthropological studies have focused on populations and ethnic groups in different parts of China [54–58]. These studies have been conducted among the Han, Dong, Yi, Chuanqin, Li, Sui and Uygur peoples, with the aim of identifying the sex, age, and skeletal characteristics of these ethnic groups. Similar studies have also been conducted in South Korea and Japan [59, 60]. The application of both forensic anthropology in HFA and forensic odontology in East Asia facilitate the identification of human remains in this region.

Japan, contributes substantively to forensic odontology on a global scale, beginning with its use in a plane crash that occurred in Japan in 1985 [61]. A total of 45 victims in this plane crash were identified using forensic odontology. Since then, the advance of forensic odontology in Japan has been rapid [62]. In 2007, the Japanese Society of Forensic Dental Science (https://jsfds.com/) was established, which provided a solid foundation for Japanese research on forensic odontology. Japan has contributed substantially to research materials in AI technology [63], postmortem computed tomography [64], and other cutting-edge forensic odontology technologies. Moreover, with the improvement of forensic systems in China and South Korea, some research in the field of forensic odontology has been conducted within these countries, mainly focusing on the dental characteristics of their respective populations [65, 66]. It is noteworthy that within this field, Japan and South Korea have been cooperating in research projects that focus on the 18-year threshold for second and third molar maturity [67], which may also contribute to HFA development in East Asia. Through the development of forensic odontology, it may become possible to distinguish the natives of different countries within East Asia more rapidly.

In the field of forensic genetics, in-depth explorations within East Asia are relevant to the HFA main focus on mass disasters. Some studies on HFA research frameworks and retrospective reflections on mass disasters have also been conducted in East Asia. These studies reflect the importance of the East Asian region within HFA research [68–70]. To cope with possible mass disasters within East Asian countries, MPS is the main research focus in these countries [71–74]. Because of geographical factors and large populations within East Asia, the prospects of applying MPS in this region are favorable, and such research also provides a foundation for conducting HFA in large-scale missing person cases.

There are other antecedents of HFA in East Asia. In the Daegu subway disaster, two medical examiners and one forensic anthropologist performed the work of confirming the identities of the victims [75]. This was an example of the implementation of HFA in East Asia, even though HFA had not been formally proposed at that time. Furthermore, in the 2011 East Japan earthquake and tsunami, forensic anthropology was applied to identify the victims [76]. In all these cases, HFA was practically implemented, indicating that there is a sound basis for applying HFA in East Asia.

**Figure 2:**
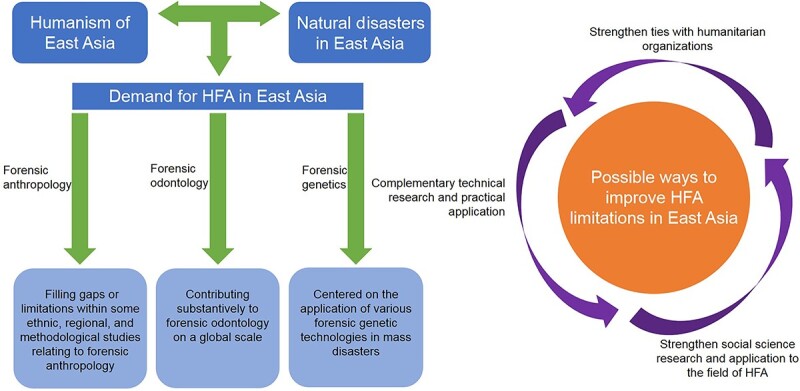
The development of humanitarian forensic action (HFA) and possible ways to improve in East Asia.

Despite the well-established history in East Asia of using forensic science for humanitarian purposes and the technological advancements that support this, at the time of this review, few studies about allied social sciences that focused on HFA or forensic humanitarian in East Asia were found. The limited number of studies could be attributed to the underestimation of the practical importance of disciplines like jurisprudence and psychology in this region. Therefore, it is necessary to cooperate with social organizations and learn from practices applied in other regions of the world so that cutting-edge tools and experiences relating to HFA can provide lessons for East Asia along with feedback from its implementation in this region [77]. Efforts to promote cooperation with various organizations and institutions worldwide may provide new directions for the development of HFA in East Asia (Figure 2).

## Conclusions

Identifying deceased persons is an important aspect of human rights and HFA, and should be the focus of humanitarian efforts. East Asia has some experience in HFA relating to the identification of missing persons. However, there is a lack of explicit discussion on humanitarian applications of forensic science and allied social sciences in the region. It is to be hoped that East Asia countries will improve the practical application of HFA, while simultaneously carrying out social surveys and social scientific research on all aspects of HFA. Meanwhile, we believe that multidimensional co-development in East Asia can interoperate in theoretical and practical experiences, and it can deepen and broaden the relevant research directions in social and natural sciences. What is more, further research in these areas will strengthen the link between HFA and clinical forensic science, establishing stronger physical and psychological care for the injured, and not just the dead. The same will also provide greater care for survivors of those who are missing and/or dead. We believe that such a trajectory can inject vitality into the development of HFA in East Asia.
